# Mapping of Human *FOXP2* Enhancers Reveals Complex Regulation

**DOI:** 10.3389/fnmol.2018.00047

**Published:** 2018-02-21

**Authors:** Martin Becker, Paolo Devanna, Simon E. Fisher, Sonja C. Vernes

**Affiliations:** ^1^Neurogenetics of Vocal Communication Group, Max Planck Institute for Psycholinguistics, Nijmegen, Netherlands; ^2^Language and Genetics Department, Max Planck Institute for Psycholinguistics, Nijmegen, Netherlands; ^3^Donders Institute for Brain, Cognition and Behaviour, Radboud University, Nijmegen, Netherlands

**Keywords:** *FOXP2*, enhancer elements, Genetic, regulation of gene expression, language, language disorders, *TBR1*

## Abstract

Mutations of the *FOXP2* gene cause a severe speech and language disorder, providing a molecular window into the neurobiology of language. Individuals with *FOXP2* mutations have structural and functional alterations affecting brain circuits that overlap with sites of *FOXP2* expression, including regions of the cortex, striatum, and cerebellum. *FOXP2* displays complex patterns of expression in the brain, as well as in non-neuronal tissues, suggesting that sophisticated regulatory mechanisms control its spatio-temporal expression. However, to date, little is known about the regulation of *FOXP2* or the genomic elements that control its expression. Using chromatin conformation capture (3C), we mapped the human *FOXP2* locus to identify putative enhancer regions that engage in long-range interactions with the promoter of this gene. We demonstrate the ability of the identified enhancer regions to drive gene expression. We also show regulation of the *FOXP2* promoter and enhancer regions by candidate regulators – FOXP family and TBR1 transcription factors. These data point to regulatory elements that may contribute to the temporal- or tissue-specific expression patterns of human *FOXP2*. Understanding the upstream regulatory pathways controlling *FOXP2* expression will bring new insight into the molecular networks contributing to human language and related disorders.

## Introduction

*FOXP2* is the first and most well-studied gene to be implicated in human speech and language skills. Heterozygous mutations of the *FOXP2* gene cause a severe speech and language disorder characterized by childhood apraxia of speech (CAS) and accompanied by expressive and receptive language problems (Vargha-Khadem et al., 1995; Watkins et al., 2002). The first *FOXP2* point mutation to be identified was a disruptive missense variant inherited by all affected members of a large three-generation pedigree, known as the KE family (Lai et al., 2001). Further mutations have been found in similarly affected individuals including missense and nonsense mutations disrupting the protein sequence (MacDermot et al., 2005; Reuter et al., 2017) and larger structural changes that affect the *FOXP2* locus such as; whole gene deletions (Feuk et al., 2006; Zeesman et al., 2006; Lennon et al., 2007; Palka et al., 2012; Rice et al., 2012; Zilina et al., 2012), chromosomal rearrangements disrupting the gene coding sequence (Lai et al., 2001; Feuk et al., 2006; Shriberg et al., 2006), and a small intragenic deletion leading to a premature stop codon (Turner et al., 2013; Reuter et al., 2017). Structural mutations near the *FOXP2* locus that do not disrupt the coding sequence have also been identified in individuals with speech and language problems (Feuk et al., 2006; Adegbola et al., 2015; Moralli et al., 2015). Some of these mutations have been hypothesized to affect non-coding regulatory elements of *FOXP2* and could therefore exert their effects by influencing expression of the gene. For example, in a child with delayed speech development, a complex structural mutation was found including a balanced inversion with a breakpoint downstream of *FOXP2* (Moralli et al., 2015). Downstream of this breakpoint, a functional enhancer was identified that was suggested to alter *FOXP2* expression and thus contribute to the disorder (Becker et al., 2015). Therefore, while it is clear that disruptions of the *FOXP2* coding region result in speech/language deficits, it may also be the case that dysregulation of this gene can contribute to similar phenotypes.

Patients with *FOXP2* mutations display alterations affecting both structural and functional brain features (Vargha-Khadem et al., 1998; Watkins et al., 2002; Belton et al., 2003; Liegeois et al., 2003, 2011). Voxel-based morphometry has identified altered gray matter density in cortical areas (including posterior superior temporal gyrus, angular gyrus, and inferior frontal gyrus), the caudate nucleus, putamen and cerebellar lobule VIIIB (Vargha-Khadem et al., 1998; Watkins et al., 2002; Belton et al., 2003). Functional activation differences during various language-related tasks have been observed in select cortical regions (including the inferior frontal gyrus), the caudate nucleus and the putamen (Belton et al., 2003; Liegeois et al., 2003; Pinel et al., 2012). The neural sites of alteration in these studies overlap with the regions of the brain where *FOXP2* is expressed, suggesting that loss of functional FOXP2 in these structures may be contributing to the neural and behavioral phenotypes.

*FOXP2* expression patterns have been characterized in the embryonic human brain, as well as in the embryonic and adult mouse brain (Ferland et al., 2003; Lai et al., 2003). These studies showed overlapping and complex patterns of expression in developing cortical, subcortical, midbrain and hindbrain regions. *FOXP2/Foxp2* expression was observed in the cortex (deep layers), basal ganglia (medium spiny neurons), thalamus, hypothalamus, inferior colliculus, medulla (inferior olive), and cerebellum (Purkinje cells). Many of the *FOXP2* positive brain regions are involved in motor control and motor learning, for example cortico-striatal-thalamic and olivo-cerebellar circuitry (Vargha-Khadem et al., 2005). However, *FOXP2/Foxp2* expression is not restricted to the brain and it is also found in the spinal cord, lungs, heart, and intestines (Shu et al., 2001; Morikawa et al., 2009). This suggests that a complex set of regulatory mechanisms control the expression of *FOXP2*. Different promoters and/or enhancers may be driving expression in neuronal versus non-neuronal tissue, directing expression in specific subsets of cells within a tissue, and ensuring it is switched on only at the appropriate developmental time points. Although these complex expression patterns of *FOXP2* were first described more than a decade ago, there is still very little known about the mechanisms by which the gene is regulated.

Transcription of *FOXP2* may be initiated at one of at least four transcriptional start sites (TSSs) (Bruce and Margolis, 2002; Schroeder and Myers, 2008). These alternative TSSs are utilized differently across different cells types and tissues; however, all but one are predicted to yield the same protein product (Bruce and Margolis, 2002; Schroeder and Myers, 2008). Thus, the role of these alternative TSSs may be to control when, where and how much FOXP2 protein is expressed, leading to cell type, tissue or developmental stage specific expression (Bruce and Margolis, 2002; Schroeder and Myers, 2008). A small number of putative enhancer elements contributing to *FOXP2* regulation have previously been identified in human and animal systems. For example, a putative enhancer element was identified in intron 8 of human *FOXP2* that was bound and regulated by the POU3F2 transcription factor (TF) (Maricic et al., 2013). A POU3F2 binding site in this enhancer was altered via a nucleotide change during the evolution of modern humans, with the ancestral allele acting as a stronger driver of reporter gene expression in experimental assays, as compared to the derived allele (Maricic et al., 2013). Three putative enhancers located upstream, downstream and within intron 2 of the zebrafish *FoxP2* coding region were shown to drive reporter gene expression *in vivo* (Bonkowsky et al., 2008). The upstream and intron 2 enhancers were directly bound by the lef1 TF and lef1 knockdown resulted in loss of *FoxP2* expression in the midbrain and hindbrain of zebrafish embryos (Bonkowsky et al., 2008). To date, no comprehensive assessment of human *FOXP2* regulatory regions has been performed.

To better understand the regulatory mechanisms controlling *FOXP2* expression in humans, we mapped putative enhancer regions at and around the *FOXP2* locus. Using chromatin conformation capture (3C) we identified genomic regions that engaged in long-range interactions with the *FOXP2* promoter, indicating possible enhancer activity. Reporter gene assays demonstrated that some of these putative enhancer regions were able to drive expression. Moreover we investigated regulation of the *FOXP2* promoter and active enhancer regions by FOXP family and TBR1 TFs. These data give insight into the upstream molecular networks and *cis* genomic regions that may influence the spatio-temporal regulation of *FOXP2*.

## Materials and Methods

### Cell Culture

HEK293, SH-SY5Y, IMR32, SK-N-AS, and KELLY cell lines were purchased from HPA Culture Collections (England, UK), SK-N-MC and PFSK1 were purchased from ATCC (Virginia, USA). The EBV transformed lymphoblast cell lines GM22671 and GM22737 were obtained from Coriell Cell Repositories (NJ, USA). All cell lines were cultured at 5% CO_2_ and 37°C in the appropriate culture media. Adherent (non-EBV) cell lines were supplemented with 10% fetal bovine serum (Invitrogen, CA, USA) and 1% penicillin/streptavidin (Invitrogen). EBV cell lines were supplemented with 15% fetal bovine serum (Invitrogen) and 1% penicillin/streptavidin (Invitrogen). The culture medium used to grow HEK293 cells was DMEM (Invitrogen), SK-N-MC and IMR32 were grown in MEM (Invitrogen) supplemented with 2 mM L-glutamine (Invitrogen), and SH-SY5Y and SK-N-AS were grown in DMEM:F12 (Invitrogen) supplemented with 2 mM L-glutamine and 1% non-essential amino acids (Invitrogen). KELLY, PFSK1, GM22671, and GM22737 were grown in RPMI 1640 media (Sigma-Aldrich, MO, USA) supplemented with 2 mM glutamine.

### *FOXP2* Expression Analysis (qPCR)

Cells were lysed in TRIzol (Invitrogen) and RNA was extracted using RNeasy Spin Columns (Qiagen, NRW, Germany). Reverse transcription was performed on 2000 ng of RNA using the High Capacity cDNA Reverse Transcription Kit (Applied Biosystems, CA, USA) using random primers, according to the manufacturer protocol. Relative expression levels were determined by real-time quantitative PCR (qPCR) using iQ SYBR Green Supermix (Bio-Rad, CA, USA). *FOXP2* mRNA was amplified using exon spanning primers with the forward primer in exon 5 (5′-ACAGCATCCTGGAAAGCAAG-3′) and reverse primer in exon 6 (5′-ATGGAGATGAGTCCCTGACG-3′). The expression of the *GAPDH* housekeeping gene was quantified using the following primers: forward 5′-AAGGTGAAGGTCGGAGTCAAC-3′, reverse: 5′-GGGGTCATTGATGGCAACAATA-3′. Differential expression (dCt) was determined by normalizing the *C*t value of *FOXP2* mRNA to the *C*t value of *GAPDH* mRNA. We then compared the *FOXP2* expression across all the cell lines, using the expression level in HEK293 cells as reference (using the ddCt method) (Livak and Schmittgen, 2001).

### Chromatin Immunoprecipitation (ChIP)

A total of 2.5 × 10^7^ cells were crosslinked with 0.5% formaldehyde for 10 min at room temperature (RT). After quenching the reaction with 125 mM glycine, the lysate was sonicated using 12 cycles of sonication (30 s on/30 s off) in a Bioruptor (Diagenode, Belgium), set to “high” frequency. 2 μl of POLR2A antibody (Diagenode, Cat# AC-055-100) was used to immunoprecipitate chromatin fragments, rotating overnight at 4°C. Purified DNA fragments were eluted in 50 μl TE. Enrichment of target DNA fragments was detected via qPCR using the iQ SYBR Green Supermix (Bio-Rad) on 2 μl sample DNA according to the manufacturer’s protocol. Primer sequences for each target fragment are listed in **Supplementary Table S1**. Statistical significance was assessed using pairwise ANOVA and *post hoc* Tukey test.

### Chromatin Conformation Capture (3C)

We used a modified 3C protocol based on Hagege et al. (2007). 1 × 10^6^ cells were crosslinked using 0.5% formaldehyde for 10 min at RT. Cells were lysed via homogenisation using a dounce tissue grinder. Chromatin was digested overnight at 37°C with 400 units of BglII (New England Biolabs, MA, USA). Following this, another 200 units of BglII restriction enzyme were added to the reaction for 1 h at 37°C. Restriction enzymes were inactivated by adding SDS (2% final) and incubation for 30 min at 37°C followed by addition of Triton X-100 (2% final) to quench SDS. Fragments were ligated using 50 units of T4 Ligase (Roche, Switzerland) in 5 ml reaction volume at 16°C overnight (ligation was performed in a high volume to favor ligation events between cross-linked DNA strands). Cross links were reversed at 65°C overnight in the presence of Proteinase K (0.5 mg/ml), and samples were then purified via phenol extraction.

Sample quantification was performed via real-time qPCR, using iQ SYBR Green (Bio-Rad) and compared against a standard curve (range 5–1000 ng) generated using human genomic DNA (Novagen). Quantification of specific ligation products was done by TaqMan real-time PCR using SsoFast Probes Supermix (Bio-Rad) according to the manufacturer’s protocol. Primer sequences are listed in **Supplementary Table S2**. The sequence of the custom made MGB-TaqMan probe was 5′-GATCTCTTAAACCACTGGGAATTCA-3′ (Applied Biosystems) and matches the sequence on chromosome 7 from nucleotide 113,732,166 to 113,732,190 (reference genome hg19). The delta *C*t value for each ligation product was calculated by subtracting the *C*t value from the average *C*t value of the same ligation product in EBV-lymphoblast cell lines. If the ligation product could not be detected in the EBV cell lines, a value of 40 was subtracted, which is equal to the lowest detected ligation product in the EBV cell lines. From the *C*t values, we calculated the relative amount of starting ligation products by raising the negative *C*t value to the power of two, which is a measure of the interaction frequency. Finally, we derived the relative interaction frequency by normalizing to the ligation product with the lowest variation across all cell lines (primer at -11 kb).

The anchor point primer and TaqMan probe were designed to match the restriction fragment that contains the first transcriptional start site (TSS1) of the *FOXP2* gene. Detection primers were designed to be complementary to the 5′ end of BglII restriction fragments so that the amplicons of the ligation products were not bigger than 250 bp. Primers were designed to match genomic fragments up to 106 kb upstream and 1,391 kb downstream of TSS1, spanning a total of 1,497 kb around the *FOXP2* gene locus. Within this genomic region, there are 428 restriction fragments produced by BglII digestion. Primers were designed for 50 of these fragments. The linear amplification range of the primers was assessed using standard curves of the PCR reaction on 3C samples with standardized concentrations (50, 125, 250, 500, 750, and 1,000 ng). Forty-five primers passed this quality control step, and they covered fragments of a total size of 218 kb. All primers are listed in **Supplementary Table S2**.

Statistical significance of the difference of 3C crosslinking frequencies to LBV-lymphoblast cell crosslinking frequencies was assessed using two-tailed student *t*-tests. *P*-values were corrected for the number of investigated interactions (45) using Benjamini-Hochberg correction.

### Luciferase Reporter Assays

Regulatory sequences were cloned from healthy human genomic DNA (Novagen) using Advantage 2 Polymerase kit (Clontech, CA, USA) according to the manufacturer’s protocol. Cloning primers are listed in **Supplementary Table S3**. PCR products were first cloned into the pCR2.1-TOPO vector using the TOPO TA Cloning Kit (Invitrogen), then subcloned into pGL4.23 (Promega) and confirmed via Sanger sequencing. Promoter elements were subcloned using SacI (5′) and XhoI (3′) restriction sites. Enhancer elements were subcloned using KpnI (5′) and XhoI (3′).

For luciferase assays, HEK293 or SK-N-MC were transfected using GeneJuice Transfection Reagent (Merck Millipore, MA, USA), with 48 ng of pGL4.23-Enhancer and 6 ng of pGL4-hRLuc-TK control plasmid (Promega, WI, USA). Luciferase enzymatic activity was determined using the Dual-Luciferase Assay System (Promega) in a TECAN Infinite 2002 plate reader (TECAN, Switzerland). The promoter and enhancer activities were each detected in three biological replicates. To test the effects of TFs on the enhancers and promoters, pGL4.23-enhancer/promoter and TF over-expression plasmids were co-transfected in HEK293 cells. FOXP1, FOXP2, and FOXP4 were overexpressed from a pcDNA3 vector (Vernes et al., 2006). TBR1 and CASK were overexpressed from a pYFP vector (Deriziotis et al., 2014). Cells were transfected with 2 ng of pGL4.23-promoter/enhancer, 2 ng of pGL4.74-hRLuc-TK and 10 ng of the individual TF plasmid.

To determine the basal activities, the relative firefly luciferase activities of the promoter/enhancer elements were compared to the firefly luciferase activity of the empty (minP) construct within the same cell lines. Statistical significance of the basal enhancer and promoter activities were assessed using pairwise ANOVA and *post hoc* Least Significant Difference (LSD) tests. The luciferase activity was compared between co-transfection of TFs or empty pcDNA4 vectors. We routinely used pcDNA4 as control because co-transfection of empty pYFP vectors with the promoter and enhancer constructs yielded comparable results (data not shown). The difference between empty plasmid and TF overexpression was assessed per individual element and the statistical significance was assessed using two-way ANOVA and *post hoc* LSD tests.

## Results

### Identification of Active *FOXP2* Promoters in Human Cell Lines

**FIGURE 1:**
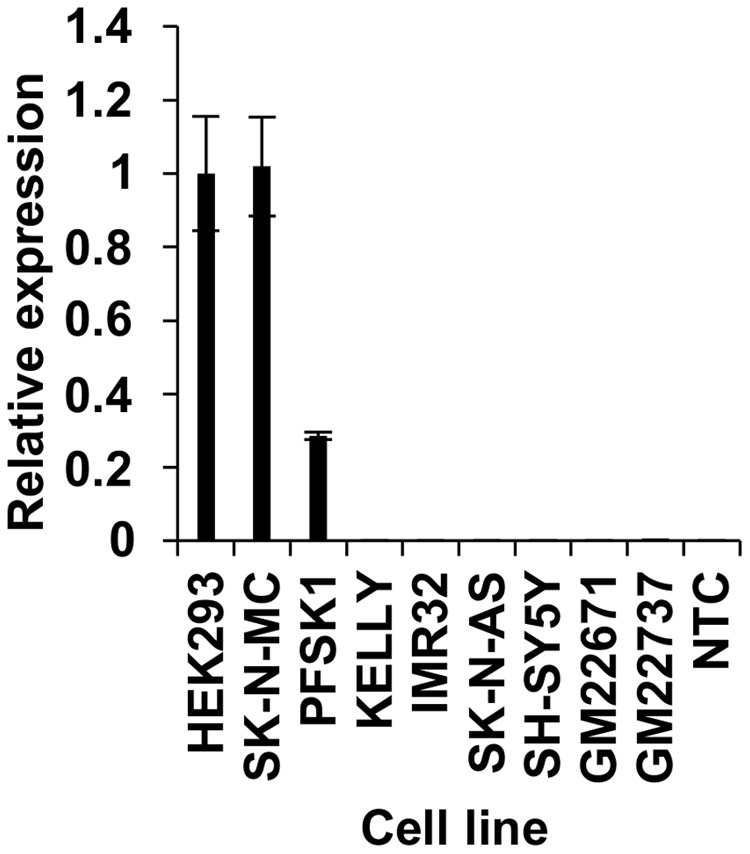
Endogenous expression of *FOXP2* in human cell lines. Endogenous expression of *FOXP2* mRNA in HEK293, SK-N-MC, PFSK1, KELLY, IMR32, SK-N-AS, SH-SY5Y, and EBV cell lines (GM22671 and GM22737) as determined by RT-qPCR. A no template control (NTC) was also included. All expression levels have been normalized to HEK293 levels as this cell line is well established to show strong expression of *FOXP2*.

To map *FOXP2* enhancer regions in human cells, it was first necessary to determine which cell lines express *FOXP2* endogenously and which promoter(s) may drive this endogenous expression. We tested endogenous expression of *FOXP2* in six human neuroblast cell lines (SK-N-MC, PFSK1, KELLY, SK-N-AS, IMR32, and SH-SY5Y), and one kidney derived cell line (HEK293). HEK293 cells are already known to endogenously express *FOXP2* and have been previously used by us and others to study its function (Vernes et al., 2006; Fu et al., 2014; Sin et al., 2015; Estruch et al., 2016). We compared *FOXP2* expression in the six neuroblast and two EBV-transformed lymphoblast cell lines (GM22671 and GM22737), relative to HEK293 cells (**Figure 1**). We included EBV cell lines here as they would be used to control for chromatin interactions in our subsequent 3C experiments. SK-N-MC cells showed comparable *FOXP2* expression levels to HEK293 cells, whereas PFSK1 expression was approximately 80% lower. None of the other neuroblast lines expressed detectable levels of *FOXP2*. Expression in EBV cell lines was detectable, but extremely low (**Figure 1**).

**FIGURE 2:**
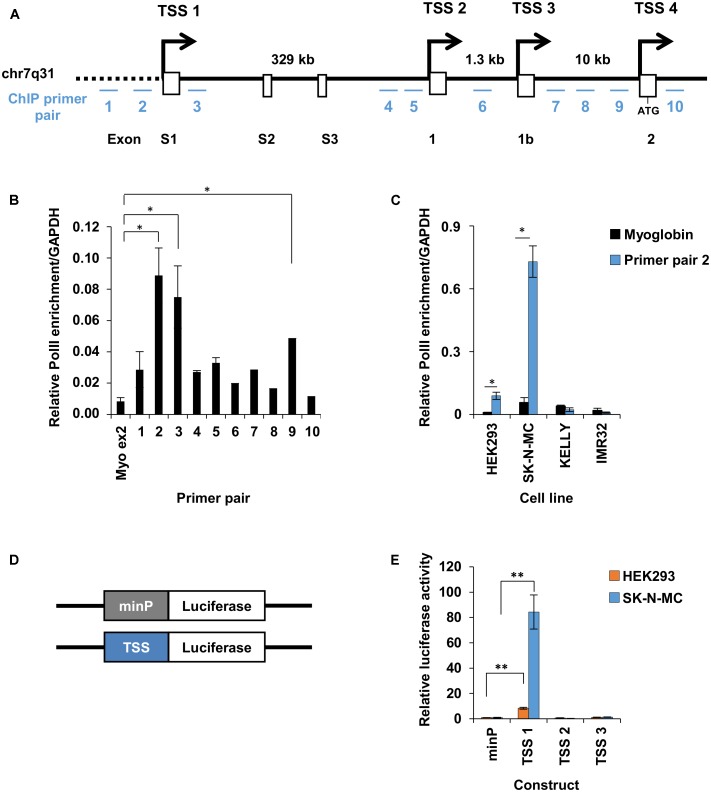
Promoter 1 of *FOXP2* drives endogenous expression. **(A)** Linear representation of the genomic location on chromosome 7q31, encompassing the 5′ end of the *FOXP2* gene. The first exons of *FOXP2*, from exon S1 to exon2 are shown as white boxes with promoters and transcriptional start sites (TSSs) at exon S1, 1, 1b and 2 indicated by arrows. The first upstream ATG start codon is located in exon 2. The relative location of the ten qPCR primer pairs used for RNAPolII ChIP are indicated by blue lines and numbered 1–10. **(B)** RNA polymerase II (RNAPolII) occupancy at the promoter regions of *FOXP2*, as measured by ChIP-qPCR. The relative PolII occupancy in HEK293 cells is shown for each primer pair and for myoglobin exon 2, which is used as a negative control. RNA-PolII occupancy is normalized to a positive control region at the *GAPDH* promoter. **(C)** Normalized PolII occupancy at TSS1 primer pair 2 and myoglobin exon 2 (negative control) is shown for the cell lines HEK293, SK-N-MC, KELLY, and IMR32. The qPCR was performed in duplicate. Significance was determined in comparison to the negative control by ANOVA followed by *post hoc* Tukey tests. ^∗^*p* < 0.05. **(D)** Schematic representation of the firefly luciferase constructs tested in **(E)**. Promoter elements (TSS) were cloned to replace the minimal promoter (minP) in pGL4.23. **(E)** Relative luciferase activity of promoter elements driving firefly luciferase gene expression in HEK293 and SK-N-MC cells. The promoter constructs were co-transfected with the pGL4.74 control plasmids expressing the renilla luciferase under the control of a herpes simplex virus thymidine kinase (HSV-TK) promoter. The firefly luciferase signal was divided by the renilla signal to derive the relative luciferase activity. The activity for each luciferase construct was normalized for the activity observed for the minimal promoter (minP). The constructs were measured in two independent experiments in a total of six biological replicates. The statistical significance of the difference between each construct and minP was determined with two-way ANOVA and *post hoc* LSD testing. ^∗^*p* < 0.05, ^∗∗^*p* < 0.001.

Transcription of the human *FOXP2* gene may be initiated from one of four previously described TSSs (**Figure 2A**), which display cell line- and tissue-specific activity (Schroeder and Myers, 2008). To identify which of these TSSs were active in HEK293 cells we first assessed RNA Polymerase II (PolII) occupancy followed by luciferase reporter assays of the putative promoter fragments. PolII occupancy is an indicator of transcription initiation and active promoter regions (Bonn et al., 2012; Core et al., 2012; Le Martelot et al., 2012). To map PolII occupancy across the four alternative TSSs we performed PolII chromatin immunoprecipitation (ChIP), followed by qPCR of the pulled down DNA fragments. Ten qPCR primers were designed, spanning the four alternative TSSs (**Figure 2A**; **Supplementary Table S1**). Primers complementary to a region that is not bound by PolII (within exon 2 of the myoglobin gene, as shown in previous studies) were used as a negative control. Significant enrichment was observed for DNA fragments spanning primer pairs 2 and 3 (adjacent to TSS1) and primer pair 9 (adjacent to TSS4), indicating that these regions may be active promoters (**Figure 2B**). Primer pair 2 was a good candidate for the location of an active promoter as it was just upstream of TSS1, which was previously shown to be active in HEK293 cells (Schroeder and Myers, 2008). As such we also tested PolII occupancy at the position of primer pair 2 in SK-N-MC neuron-like cells that endogenously express *FOXP2*, and two neuron-like cell lines that do not express endogenous *FOXP2* (KELLY and IMR32 cell lines). PolII was strongly enriched in SK-N-MC cells (∼8-fold stronger enrichment compared to HEK293 cells), but was not enriched in KELLY or IMR32 cells (**Figure 2C**).

Since there was PolII enrichment just upstream of TSS1 in the two *FOXP2* positive cell lines tested (HEK293 and SK-N-MC), we went on to determine if TSS1 was an active promoter and could drive reporter gene expression in both cell lines. We cloned a 1,791 bp fragment that spanned 1,547 bp upstream of TSS1 (including the primer regions 1 and 2) and 244 bp downstream (including part of the 5′UTR of *FOXP2* but excluding the start codon). This fragment was placed upstream of a firefly luciferase reporter gene to act as its promoter (**Figure 2D**). We also compared expression from constructs containing comparable fragments spanning TSS2 (∼4.1 kb fragment) and TSS3 (∼1 kb fragment). These regions had not shown PolII occupancy in HEK293 cells, suggesting they are inactive promoters and thus they are not expected to be able to drive luciferase expression above the level of the baseline control. The baseline control was an equivalent construct containing a minimal promoter region (minP) (**Figure 2D**). Details of the cloned elements are given in **Table 1**. Measurement of relative luciferase activity recapitulated the pattern observed for PolII occupancy. The fragment spanning TSS1 resulted in significantly stronger reporter gene expression in both HEK293 and SK-N-MC lines, but the effect was ∼8-fold stronger in SK-N-MC cells (**Figure 2E**). The ability of TSS1 to drive expression in HEK293 cells is consistent with previous reports (Schroeder and Myers, 2008). The fragments spanning TSS2 and TSS3 did not increase luciferase activity above the levels of the minP baseline control, confirming them as inactive regions in these cell lines. These data suggest that TSS1 represents an active *FOXP2* promoter region in HEK293 and SK-N-MC cell lines. As such we went on to use TSS1 to identify putative *FOXP2* enhancers that make contact with this promoter region.

**Table 1:** *FOXP2* cloned promoter (TSS) sequences used for reporter assays.

Element	Size (bp)	Start (hg19)	End (hg19)	Mean conservation (phastCons)
TSS1	1,791	113,724,817	113,726,609	0.34
TSS2	4,104	114,051,220	114,055,324	0.78
TSS3	1,006	114,055,454	114,056,459	0.93

### Identification of Putative *FOXP2* Enhancers via Chromatin Conformation Capture (3C)

**FIGURE 3:**
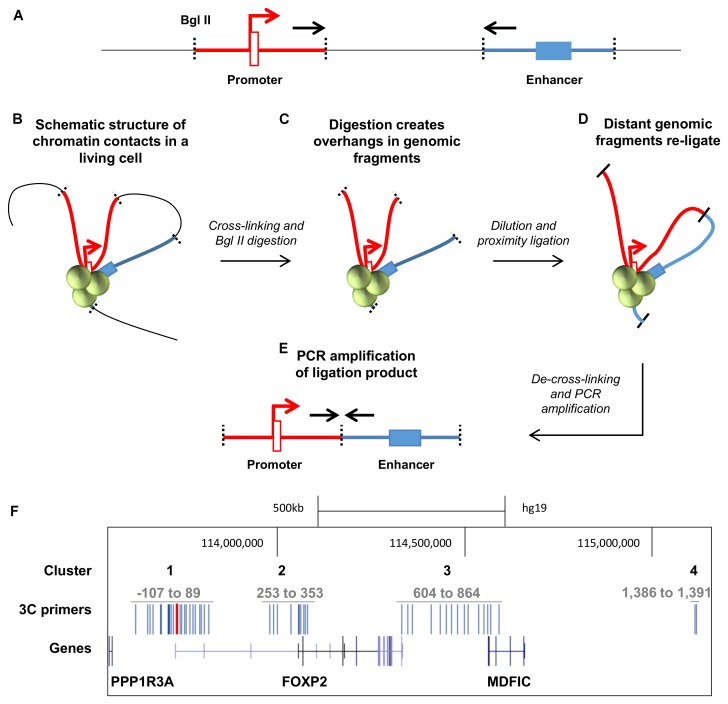
Chromatin conformation capture (3C) design for the *FOXP2* gene locus. **(A)** Possible enhancer elements (blue blocks) are distant to their target promoters (red block and arrow) on a linear representation of the genome. Specific target primers (black arrows) are designed at BglII restriction sites (black dotted lines). **(B)** In living cells the genome is folded by proteins (green spheres) and active enhancers contact their target promoters. The chromatin packed genome is cross-linked in this state and digested with BglII restriction enzymes. **(C)** The genomic fragments and DNA overhangs remain in physical proximity. The DNA is diluted to favor proximity ligations between these genomic fragments. **(D)** Genomic fragments that were distant in the linear genome are ligated to form one short DNA ligation product. **(E)** The primers that were distant in the genome are now close enough to amplify from the ligation product. **(F)** The genomic location of the *FOXP2* gene. The gene structure of *FOXP2* and the neighboring genes *PPP13RA* and *MDFIC* are shown in dark blue. The anchor point primer is located at the distal promoter 1 and shown in red. The 3C target primers (depicted in blue) were designed to cluster at four locations of the gene locus: Cluster 1 is around the anchor point at TSS1. Cluster 2 is clustered around the downstream promoters TSS2-4. Cluster 3 is located at the downstream intergenic region between the *FOXP2* 3′UTR and *MDFIC*. Cluster 4 is downstream of the *MDFIC* gene. *FOXP2* TSS1 is located at nucleotide 113,726,365 of the human genome in chromosome 7 (hg19). The 3C primers (blue bars) were designed just upstream of the predicted BglII restriction sites.

Identifying enhancer regions is challenging as they can be found at distances of up to 1 Mb from the genes they regulate (Pennacchio et al., 2013). However, in order to contribute to gene regulation, enhancers loop around to make physical contact with promoter regions, facilitating the formation of protein complexes that drive gene expression. Chromatin conformation capture (3C) is a method that allows these three-dimensional contacts to be identified (**Figures 3A–E**). In brief, the method involves cross linking cells to preserve endogenous three-dimensional structures (**Figure 3B**), digesting the DNA using a restriction enzyme with evenly spaced digestion sites (in our case BglII) to create overhangs in the looped DNA (**Figure 3C**), and directly ligating these previously physically distant overhangs to each other (**Figure 3D**). After reversal of cross links and DNA extraction, the ligated ends can be detected via PCR amplification of primers that span the two regions (i.e., promoter and putative enhancer region) (**Figure 3E**). The presence of a PCR product demonstrates that the two regions were in close physical contact in the endogenous cell line.

Because TSS1 was strongly active in both HEK293 and SK-N-MC cell lines, we identified the putative enhancer elements that made contact with this promoter region. To this end, we designed a Taqman probe to the 3′-end of the BglII restriction fragment that contains TSS1 (**Figures 3A,E**). We then used reverse primers within 45 restriction fragments (hereafter called ‘3C fragments’), spanning four genomic regions that were considered likely to contain enhancers regulating *FOXP2* (**Figure 3F**). The majority of enhancers can be found within 200 kb distance of a TSS (Li et al., 2013) and cluster 1 targets enhancers in a 200 kb window around TSS1, which spans the intergenic region between *FOXP2* and *PPP1R3A* and the first intron of *FOXP2* (**Figure 3F**: Cluster 1). Cluster 2 targets a 100 kb window around the alternative start sites TSS2-4 of *FOXP2* (**Figure 3F**: Cluster 2). Cluster 3 targets the intergenic region between the *FOXP2* and the *MDFIC* gene including the 3′-end of *FOXP2* and the 5′-end of *MDFIC* (including exons 1–3 and intron 4) (**Figure 3F**: Cluster 3). This cluster includes a previously reported functional enhancer that was suggested to regulate *FOXP2* (Becker et al., 2015). In addition, two target primers were designed to an intronic region 460 kb downstream of the *MDFIC* gene (**Figure 3F**: Cluster 4). We performed 3C in the seven human cell lines described above, along with two EBV-transformed lymphoblast cell lines which acted as a baseline reference since *FOXP2* expression is low in EBV cells (**Figure 1**) and we do not expect neuronal enhancers to be active in these blood-derived cells.

**FIGURE 4:**
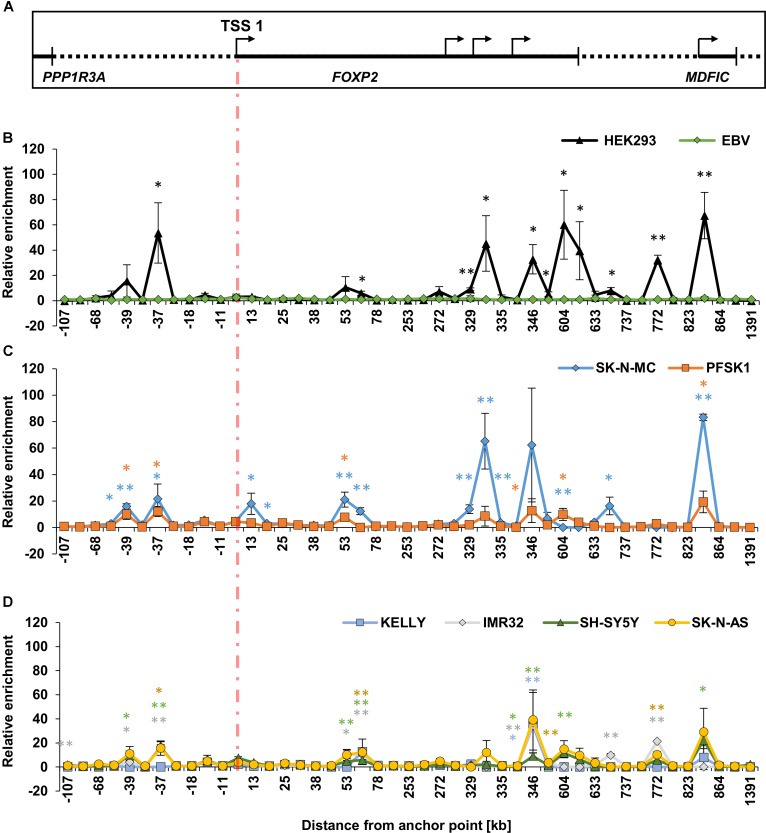
Chromatin conformation capture (3C) interaction landscape at the *FOXP2* gene locus. Chromatin interactions at the *FOXP2* gene measured by chromatin conformation capture (3C). **(A)** Schematic representation of the genomic locus of *PPP1R3A*, *FOXP2*, and *MDFIC*. The indicated promoters (TSSs; arrows) and 3′UTR (black bar) align with the data points of the following 3C graphs. **(B–D)** The relative interaction frequencies of genomic fragments to the promoter anchor point are shown according to their distance to the TSS. **(B)** Chromatin interactions in HEK293 and EBV cell lines. **(C)** Interactions in neuronal-like *FOXP2* positive cell lines SK-N-MC and PFSK1. **(D)** Interactions in neuronal-like *FOXP2* negative cell lines KELLY, IMR32, SH-SY5Y, and SK-N-AS. For all sections, the cross-linking frequencies for each target primer were normalized to the cross-linking frequency of the target primer at –11 kb (internal normalization). The cross-linking frequency for each target primer was normalized to the average cross-linking frequency of three measurements in two EBV lymphoblast cell lines (between sample normalization). All interactions were determined in three biological replicates. Significance was calculated using two-tailed student *t*-tests. *P*-values were corrected for the amount of tested genomic fragments using Benjamini-Hochberg correction. ^∗^*p*-value < 0.01; ^∗∗^*p*-value < 0.001

Because the TSS1/promoter 1 is active in HEK293 cells and they strongly express endogenous *FOXP2*, we first assessed the interactions of the 45 different 3C fragments with TSS1 in these cells, compared to the ‘baseline’ EBV cell lines. We mapped the relative enrichment of each fragment in HEK293 cells by first normalizing each interaction to a promoter-adjacent fragment, which is in close linear sequence proximity to promoter 1 (located at -11 kb) (**Figure 4A**). We chose this fragment because it showed the lowest variation across all cell lines, likely caused by interactions facilitated by linear sequence proximity. We further normalized to the interactions in the EBV control. We compared the interactions in HEK293 cells with the EBV baseline using two-sided *t*-tests and adjusted for multiple testing by Benjamini-Hochberg correction. Eleven fragments (-37, 70, 329, 330, 346, 353, 604, 621, 706, 772, and 843) displayed significant enrichment in HEK293 cells (**Figure 4B**, **Table 2** and **Supplementary Table S4**), suggesting that they physically interact with TSS1/promoter 1.

**Table 2:** Significant 3C fragment interactions in *FOXP2* expressing cell lines.

	HEK293		SK-N-MC		PFSK1	
3C-fragment	Mean interaction frequency	adjusted *p*-value	Mean interaction frequency	adjusted *p*-value	Mean interaction frequency	adjusted *p*-value
-59	3.99	1.23E-01	2.84	**4.33E**-**03**	0.99	4.61E-01
-39	15.69	5.50E-02	15.95	**3.35E**-**06**	10.01	**5.72E**-**03**
-37	53.64	**3.19E**-**03**	21.55	**6.97E-03**	12.10	**2.27E**-**03**
13	3.13	1.08E-01	17.86	**4.09E**-**03**	3.44	1.15E-01
17	0.62	2.93E-01	3.06	2.47E-03	0.70	4.86E-01
53	10.53	6.25E-02	21.08	**3.24E**-**04**	7.69	**1.06E**-**03**
70	5.93	**1.97E**-**03**	12.33	**1.13E**-**04**	3.10	3.59E-01
329	9.22	**2.68E**-**04**	13.77	**3.46E**-**04**	1.99	5.50E-01
330	45.28	**4.86E**-**03**	65.26	**5.37E**-**04**	8.56	1.10E-01
335	2.97	4.79E-02	3.65	**5.63E**-**04**	0.98	9.06E-01
342	0.70	1.46E-01	1.14	3.02E-01	0.19	**3.66E**-**03**
346	32.82	**1.57E**-**03**	62.29	1.68E-02	12.77	5.22E-02
353	5.24	**3.01E**-**03**	6.79	3.37E-02	2.05	6.79E-02
604	60.15	**3.55E**-**03**	0.00	**1.77E**-**23**	9.88	**8.01E**-**03**
621	39.55	**9.72E**-**03**	0.67	2.56E-01	3.94	1.46E-02
706	7.94	**1.26E**-**03**	16.26	**2.43E**-**03**	0.88	3.65E-01
772	32.41	**3.18E**-**06**	1.00	1.00E+00	2.80	3.60E-01
843	67.39	**4.68E**-**04**	83.28	**1.68E-08**	19.30	**7.16E**-**03**

*Bold represents significant p-values (p < 0.01).*

Unlike HEK293 cells, SK-N-MC and PFSK1 cells are neuron-derived cell lines, but all three cell lines endogenously express *FOXP2* (**Figure 1**). As such, we mapped the relative enrichment of the 45 3C fragments in SK-N-MC and PFSK1 cells to determine if the pattern observed in HEK293 cells was conserved in these other *FOXP2* expressing neuronal cell lines. Thirteen regions in SK-N-MC and six regions in PFSK1 cells were significantly enriched (**Figure 4C** and **Table 2**). A number of these regions were shared between SK-N-MC and PFSK1 cells (region -39, -37, 53, 604, 843), between SK-N-MC and HEK293 cells (-37, 70, 329, 330, 604, 706, 843), between PFSK1 and HEK293 cells (-37, 604, 843), or between all three cell lines (-37, 604, 843) and thus may represent common enhancer regions used in cells endogenously expressing *FOXP2*. Two regions (-39 and 53) were significantly enriched in both neuronal cell lines, but not in HEK293 cells, which may point to neuronal specific activity. In total we detected eighteen chromatin interactions between genomic regions and TSS1/promoter 1 in *FOXP2* expressing cell lines, which were not observed in lymphoblast cells (EBVs).

To determine if these chromatin interactions were unique to *FOXP2* expressing cell lines we also measured the 3C relative enrichment in four neuroblast cell lines for which endogenous FOXP2 could not be detected; KELLY, IMR32, SH-SY5Y and SK-N-AS cells (**Figure 4D** and **Supplementary Table S4**). Many of the peaks of enrichment observed in the *FOXP2* positive cells lines (**Figures 4B,C**), were also present in the *FOXP2* negative cells (**Figure 4D**). However, overall, the enrichment observed in *FOXP2* negative cells was much weaker than in *FOXP2* positive cell lines.

### *In Silico* Prediction of Enhancer Elements within 3C Fragments

The 3C fragments at -37, 330, and 843 showed the strongest and most consistent enrichment in the *FOXP2* positive cell lines (**Figures 4B,C**). However, since these interactions were detected in human cell lines, we used publicly available chromatin interaction data (Hi-C) of human post-mortem samples (Won et al., 2016; Zhang et al., unpublished) to evaluate the interactions in fetal and adult brain tissue. We visualized chromatin interactions in the browser application supplied by YUE lab (Wang et al., unpublished). In fetal cortical tissue, chromatin interactions between TSS1 and 3C fragments -37, 330, and 843 can be observed (**Supplementary Figure S1**). In adult cortical tissue, these interactions are weaker and comparable to the background dynamics of chromatin interactions at this genomic locus. Thus, the public data confirms the presence of the 3C interactions with TSS1 and suggests that they are present in the developing human brain.

Because our 3C approach utilized the BglII restriction enzyme, it produced 3C fragments of between 1 and 10 kb. Enhancer regions are typically only a few hundred nucleotides in size (Adam et al., 2015), making it necessary to narrow down the likely position of the putative enhancers within each enriched 3C fragment. To identify putative enhancer elements within these large regions we used functional genomic annotations imputed from twelve epigenetic marks (eleven histone modifications and DNase hypersensitivity) mapped in human neuronal tissue by the Roadmap Epigenomics Project (Bernstein et al., 2010; Ernst and Kellis, 2015; Roadmap Epigenomics et al., 2015). Active neuronal enhancers were predicted by strong histone-3-lysine-4-monomethylation (H3K4Me1) and histone-3-lysine27-acetylation (H3K27Ac) within the 3C fragments at 330 and 843 kb (**Table 3**). The 3C fragment at -37 kb encompassed a weak neural enhancer, predicted by strong H3K4Me1 and weak H3K27Ac. In some neuronal Roadmap Epigenomics samples, parts of the same fragment were annotated as an active TSS, predicted by an absence of H3K4Me1 and strong histone-3-lysine-9-acetylation (H3K9Ac). In this way, we identified smaller putative enhancer regions within the 3C-interacting fragments.

**Table 3:** Epigenetic marks within 3C fragments.

3C fragment	Positive enrichment (*p*-value)		Roadmaps neuronal functional element (hg19 coordinates)
	HEK293	SK-N-MC	
-37	<0.001	N.S.	Active TSS (113688600–113688799), Weak enhancer (113688002–113689199)
330	<0.008	<0.001	Active enhancer (114057400–114057799)
843	<0.001	<0.001	Active enhancer (114569600–114570599)

### Putative Enhancer Activity Demonstrated via Reporter Assays

**FIGURE 5:**
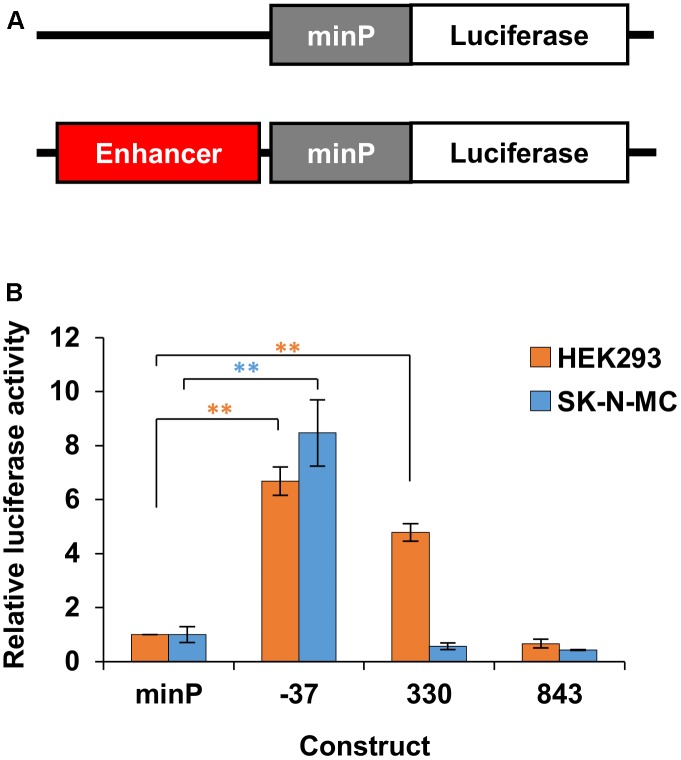
Enhancer activity in HEK293 and SK-N-MC. **(A)** The identified putative enhancer elements were cloned upstream of the minimal promoter in a firefly luciferase reporter gene construct. **(B)** Relative luciferase activity of enhancer elements driving firefly luciferase gene expression in HEK293 and SK-N-MC cells. The luciferase constructs containing the enhancers and the control vector with the minimal promoter (minP) only were each co-transfected with a control vector expressing the renilla luciferase under the control of the HSV-TK promoter. The firefly luciferase signal was divided by the renilla signal to derive the relative luciferase activity. The activity for each luciferase construct was normalized for the activity observed for the minimal promoter (minP). The constructs were measured in two independent experiments in a total of six biological replicates, with the exception of element 843 in SK-N-MC cells, which was measured in one experiment in three biological replicates. The significance of the difference between each construct and minP was determined with two-way ANOVA and *post hoc* LSD testing. ^∗∗^*p* < 0.001.

Having identified putative enhancer regions via 3C and *in silico* analysis, we next aimed to verify the ability of these regions to enhance gene expression using reporter assays. We cloned the putative enhancer regions (**Table 4**) upstream of a minimal promoter (minP) and firefly luciferase reporter gene (**Figure 5A**). We compared reporter gene expression from these constructs with equivalent constructs lacking an enhancer element (**Figure 5A**). The putative enhancer located within 3C fragment -37 was able to strongly and significantly increase reporter gene expression in both HEK293 and SK-N-MC cell lines (**Figure 5B**). The putative enhancer located within 3C fragment 330 strongly and significantly drove expression in HEK293 cells, but not SK-N-MC cells. Finally, the putative enhancer located within 3C fragment 843 was not able to enhance reporter gene expression in either cell line (**Figure 5B**).

**Table 4:** Cloned putative enhancer regions.

Element	Size (bp)	Start (hg19)	End (hg19)	Mean conservation in 100 vertebrates (phastCons)
Enhancer -37	774	113688009	113688782	0.023
Enhancer 330	1801	114056845	114058646	0.891
Enhancer 843	3958	114568454	114572411	0.111

### Regulation of the *FOXP2* Promoter and Enhancers by Transcription Factors

Herein we have shown that the *FOXP2* TSS1/promoter 1 is active in HEK293 and SK-N-MC cells, and identified putative enhancer regions that make physical contact with this promoter and can enhance gene expression in reporter assays (enhancer -37 and 330). In a previous study we also identified a putative enhancer element downstream of *FOXP2* at position 815 (named ‘*Element 1*’ in that study), which was disrupted in a child with language impairment and could drive gene expression in both HEK293 and SK-N-MC cell lines (Becker et al., 2015). Given that little is known about the TFs that regulate the expression of *FOXP2*, we set out to determine if these three enhancer regions or the *FOXP2* promoters themselves could be regulated by selected TFs. TFs often regulate their own expression in positive or negative feedback loops (Crews and Pearson, 2009; Bhatia et al., 2013), and thus we first tested if FOXP2 protein was capable of auto-regulation via interaction with TSS1/promoter 1 or enhancers -37, 330, and 815. Given its close homology to *FOXP1* and *FOXP4* and their overlapping expression patterns, we also tested these other FOXP family members for their ability to regulate gene expression via interaction with these regions. Lastly, the *TBR1* TF displays an overlapping expression pattern with *FOXP2* and these proteins are known to interact to regulate gene expression (Deriziotis et al., 2014). As such we also asked if TBR1 was able to regulate *FOXP2* expression, either in the presence or absence of CASK, a co-activating factor that interacts with TBR1 to regulate genes underlying cortical development (Hsueh et al., 2000; Deriziotis et al., 2014).

**FIGURE 6:**
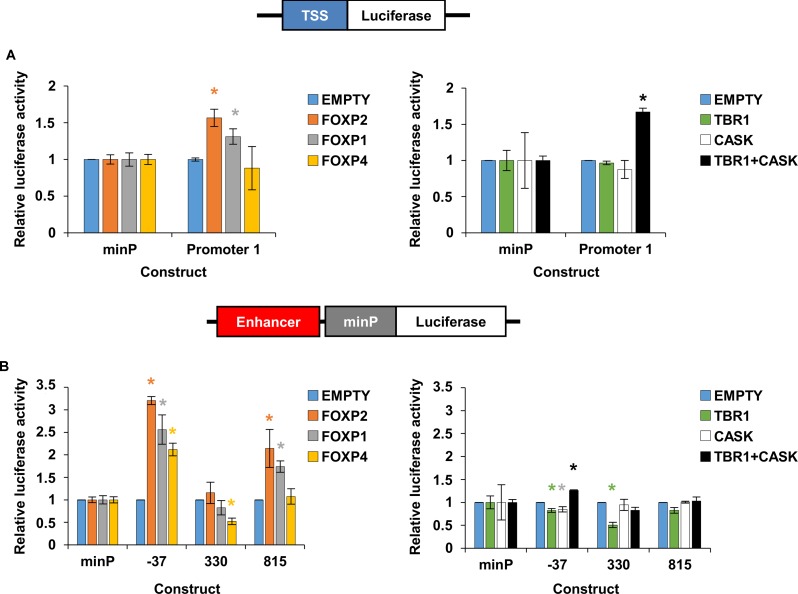
Regulation of *FOXP2* promoter and enhancers by transcription factors. Relative luciferase activity of promoter elements **(A)** or enhancer elements **(B)** after co-transfection with FOXP2, FOXP1, FOXP4, TBR1, CASK and the combination of TBR1 with CASK in HEK293 cells. The renilla luciferase control plasmid was used for internal normalization. An empty pcDNA4 vector (EMPTY) that did not express any transcription factors was included as a control. Note that HEK293 cells have been shown to endogenously express FOXP1, FOXP2, FOXP4, and CASK, but not TBR1 (Thul et al., 2017). The firefly luciferase signal was divided by the renilla signal to derive the relative luciferase activity. The activity for each luciferase construct was normalized to the co-transfection condition using an empty plasmid. Each combination was tested in three biological replicates. The statistical significance of the TF overexpression effect was determined with two-way ANOVA and *post hoc* LSD testing for each construct. ^∗^*p*-value < 0.05.

A small but significant increase in reporter gene expression was observed when either FOXP2 or FOXP1 were introduced into cells alongside the TSS1/promoter 1 fragment (**Figure 6A**, left panel). No increase was observed when TBR1 or CASK alone were introduced, but when these co-factors were introduced together to the same cells, they resulted in a significant increase in the reporter gene expression driven by the TSS1/promoter 1 (**Figure 6A**, right panel). From these reporter gene assays, we can conclude that FOXP2, FOXP1 and TBR1-CASK can drive gene expression from the *FOXP2* TSS1/promoter 1 region.

To determine if these TFs could also drive gene expression by interacting with the three identified *FOXP2* enhancer regions, we transfected the same set of TFs into cells alongside reporter constructs containing enhancer region -37, 330 or 815 upstream of a minimal promoter and the firefly luciferase reporter gene (**Figure 6B**). Co-transfection with FOXP2 or FOXP1 led to a significant increase in expression driven by enhancers -37 and 815 (**Figure 6B**, left panel). FOXP4 significantly increased gene expression when enhancer -37 was present and led to a small but statistically significant decrease in expression in the presence of enhancer 330 (**Figure 6B**, left panel). TBR1 or CASK alone led to a small but statistically significant reduction in expression driven by enhancer -37 and TBR1 alone also reduced gene expression via enhancer 330 (**Figure 6B**, right panel). However, co-transfection of TBR1 and CASK resulted in a significant increase in reporter gene expression, driven by enhancer -37 (**Figure 6B**, right panel). Thus, FOXP2, FOXP1, FOXP4, and TBR1-CASK can all influence gene expression by interacting with one or more of the identified *FOXP2* enhancer elements.

## Discussion

Herein we describe the first systematic exploration of enhancer regions of human *FOXP2*. Our data show that TSS1 is an active promoter in human cell lines and identify eighteen different genomic regions that are in physical contact with this promoter across the cell lines tested, which differ in origin and endogenous *FOXP2* expression. We show that two of these regions (located 37 kb upstream and 330 kb downstream of TSS1, respectively) are able to enhance gene expression in reporter assays. We also show that these regions, along with a previously identified putative enhancer (located 815 kb downstream of TSS1) and the TSS1 promoter, can all be regulated by FOXP family members. Promoter 1 and the 37 kb upstream enhancer are also regulated by the TBR1/CASK complex.

Of the three TSSs tested via reporter assays, only TSS1 was active in HEK293 and SK-N-MC cell lines and as such we used this promoter to identify physical interactions and map enhancer regions. TSS2 and TSS3 were not significantly different from the baseline (minimal) promoter. This is consistent with what has been previously observed in human cell lines (Schroeder and Myers, 2008). Thus, TSS1 seems to be a key promoter of *FOXP2* expression, both in neuronal (SK-N-MC) and non-neuronal (HEK293) human cells. However, these experiments were carried out using *in vitro* cell lines and as such we could not capture the full range of conditions under which these TSSs would be functional. Indeed, TSS2-3 have been shown to be active in human tissues including the basal ganglia, trachea and colon (Schroeder and Myers, 2008).

We compared the physical interactions of enhancers in neuronal and non-neuronal cell lines, with and without endogenous *FOXP2* expression. Cells that had endogenous *FOXP2* expression (HEK293, SK-N-MC, and PFSK1) showed comparable patterns despite one of the cell lines being non-neuronal. Only two regions (located 39 kb upstream and 53 kb downstream) showed significant interaction in the neuronal *FOXP2* positive cell lines that were not seen in the non-neuronal *FOXP2* positive line. This could point to a neuron specific function for these putative enhancers, however, further experimental work such as testing in more neuronal and non-neuronal cells/tissues and functional validation of these enhancers is required to demonstrate if this is indeed the case. When comparing all the *FOXP2* positive and *FOXP2* negative cell lines, many of the same regions were significantly enriched, however, the magnitude of the relative enrichment was substantially lower in *FOXP2* negative cells. Thus it may be that in cells that do not express *FOXP2*, these enhancer regions make contact with TSS1 less frequently, or that the interactions are less stable, possibly due to a lack of stabilizing TF complex formation (Ong and Corces, 2014; Allen and Taatjes, 2015). A limitation of this study is that the 3C interactions were obtained in immortalized human cell lines. However, Hi-C undertaken in human fetal brain supported contacts between TSS1 and the key regions we identified (3C fragments: -37, 330, and 815) (Wang et al., unpublished), suggesting that these cell line-identified interactions are also present in the developing human brain.

Two strong enhancers were identified within the 3C fragments located 37 kb upstream and 330 kb downstream of *FOXP2* TSS1. Enhancer 330 is evolutionarily conserved across vertebrates, whereas enhancer 37 shows low conservation, even across primates. Evolutionary conservation can be regarded as the result of functional constraint on sequence variety (Ureta-Vidal et al., 2003; Miller et al., 2004) and highly conserved enhancers are likely to drive target gene expression across related species. Thus, we predict that the enhancer region located 330 kb downstream of TSS1 is also likely to be a functional enhancer in non-human species where it is highly conserved such as primates, mice, and songbirds. Indeed, pronuclear injection of enhancer 330 into mice results in forebrain expression of a reporter gene at embryonic day E11.5 (Visel et al., 2007). This demonstrates that enhancer 330 is able to drive expression in the mouse brain, and given its conservation, points to a possible role in driving early forebrain *FOXP2* expression in humans. The presence of an evolutionary non-conserved enhancer at 37 kb upstream may suggest that this regulatory region has changed on the human lineage. Comparative studies of the ability of this enhancer to drive expression would show if it has gained function over recent evolution. If so, determining which specific aspects of *FOXP2* expression it regulates could give insight into how evolution may have shaped the role of this gene through modification of its expression pattern.

We showed that FOXP2 is capable of auto-regulation, increasing expression via interactions with promoter 1 (TSS1) and the enhancers located 37 kb upstream and 815 kb downstream of TSS1. Positive auto-regulatory loops such as this can enable rapid amplification of a protein product to maintain expression at stable plateau levels (Bateman, 1998) and auto-regulation has been described for developmental (Bateman, 1998; Di Gennaro et al., 2013; Mead et al., 2013) and neurodevelopmental TFs (Meredith et al., 2009). Thus, our data suggest that once expressed, FOXP2 contributes to maintenance of its own expression via positive auto-regulation. FOXP1 showed the same capacity for regulation as FOXP2, increasing expression via interaction with promoter 1, enhancer -37 and enhancer 815. FOXP4 only drove expression from enhancer -37. Thus *FOXP2* is also regulated by other members of the FOXP subfamily. Although they have several sites of independent expression (such as in different layers of the cortex), it is notable that FOXP2 is co-expressed with FOXP1 in the thalamus, hypothalamus and basal ganglia of multiple species (Takahashi et al., 2008b; Mendoza et al., 2015). FOXP4 expression is strong during development and overlaps with FOXP2 in the ganglionic eminences, cortical plate and thalamus (Takahashi et al., 2008a). In adult brains of multiple species FOXP4 is co-expressed with FOXP2 in Purkinje cells, thalamus and the inferior olives (Takahashi et al., 2008a; Mendoza et al., 2015). Given that FOXP1 and FOXP4 are capable of heterodimerisation with each other as well as with FOXP2 in order to regulate gene expression (Li et al., 2004; Sin et al., 2015), these proteins may contribute to the auto-regulatory mechanisms of FOXP2 in the co-expressed brain regions and/or regulate *FOXP2* independently.

In our assays, we also showed that the combination of the TBR1 TF and its cofactor CASK were able to regulate expression from promoter 1 and enhancer -37. TBR1 or CASK alone did not increase gene expression in these experiments suggesting that it was the combined action of this protein complex that resulted in regulation. The expression pattern of *TBR1* partially overlaps with *FOXP2*, both being found in the olfactory bulb and neurons in the developing cortical plate and adult cortical layer VI (Hevner et al., 2001; Hisaoka et al., 2010; Willsey et al., 2013). CASK has been shown to be important for TBR1 activity and the TBR1/CASK complex is involved in regulation of genes underlying cortical development, such as the neural extracellular matrix gene *RELN* and the NMDA receptor subunit 2b (*GRIN2b*) (Wang et al., 2004; Hsueh, 2006). It was previously shown that FOXP2 and TBR1 are able to interact to co-regulate gene expression (Deriziotis et al., 2014). Taken together these data suggest that TBR1 has the capacity to modulate *FOXP2* expression levels (via interaction with CASK) however, demonstration of TBR1 binding to these regions, e.g., via ChIP experiments in brain tissue, would be valuable to show direct regulatory effects.

In the current study we were only able to explore a limited number of fragments in and around the *FOXP2* locus, given the candidate 3C-qPCR approach. Enhancers may be located megabases from the promoters they regulate. While we could demonstrate physical interactions as far as ∼0.85 Mb from TSS1, there may also be enhancers located further upstream or downstream of TSS1 that are important for *FOXP2* expression. The genome is partitioned into topological associated domains that are evolutionarily conserved, stable during development and thought to define regulatory and physically interacting genomic units (Dixon et al., 2012). Dynamic enhancer contacts can take place within these topological domains, but not across their topological boundaries (Dixon et al., 2012). All 3C fragments investigated in this study were located within the topological domain that contains the *FOXP2* gene. However, the domain spans ∼2.4 Mb (GRCh37/hg19 chromosomal coordinates: chr7:113382764–115772764) and thus contains sequence that was not covered in our 3C experiments, including the *PPP1R3A* gene upstream of *FOXP2* and the downstream region encompassing the *MDFIC* and *TFIC* genes. Because we used a candidate approach to map physical interactions with TSS1 our data represent a systematic, yet incomplete mapping of *FOXP2* enhancers. They should therefore be considered a starting point for further enhancer identification to allow a complete understanding of the spatio-temporal regulation of *FOXP2*, for example through an extension of the 3C approach, or the application of high-throughput 3C-sequencing based methods (Nagano et al., 2013; Mifsud et al., 2015). Although not a saturation study of the FOXP2 topological domain, this work gives insight into the regulatory elements that may be controlling *FOXP2* expression, and their transcriptional regulation. Defining the regulatory mechanisms governing *FOXP2* and the molecular pathways upstream of this gene are crucial to elucidate its contributions to the development and functions of neuronal circuitry underlying speech and language.

## Author Contributions

SCV, SEF, and MB conceived the study. MB, PD, and SCV acquired and analyzed the data. SCV wrote the article. SCV, MB, PD, and SEF revised and edited the manuscript.

## Conflict of Interest Statement

The authors declare that the research was conducted in the absence of any commercial or financial relationships that could be construed as a potential conflict of interest.

## Supplementary Material

FIGURE S1 Chromatin interactions obtained from publicly available Hi-C data in neuronal tissue samples. Chromatin interactions at chromosome 7 between nucleotide 113350000 and 114640000 (hg19) determined via Hi-C were visualized in the “Compare Hi-C” browser application (http://promoter.bx.psu.edu/hi-c/compare.php; last accessed on 05.01.2018) supplied by the YUE lab (Wang et al., unpublished). The middle panel displays the location of *FOXP2* and the neighboring genes *PPP1R3A* and *MDFIC*. The position of the *FOXP2* promoters at TSS1, TSS2, and TSS3 and the putative enhancers -37, 330, 815, and 843, are annotated. The top panel shows the heat map of chromatin interactions in a sample of human fetal cortical plate tissue (Won et al., 2016) and the bottom panel shows the heat map of chromatin interactions in a sample of human adult cortex tissue (Zhang et al., unpublished). Color intensity indicates the strength of the measured chromatin interaction between two distant regions at a resolution of 10 kb. In the fetal cortical plate, TSS1 of *FOXP2* shows stronger interactions to genomic regions at enhancers -37, 330, and 843 than to other regions in this genomic locus (black arrows). In the adult cortex, the chromatin interactions between TSS1 and these regions were generally weaker (black arrows). The dashed lines indicate the interaction between 10 kb blocks containing the elements of interest.

TABLE S1 ChIP-qPCR Primers.

TABLE S2 Primers used for 3C-qPCR.

TABLE S3 Primers used to clone promoter and enhancer regions.

TABLE S4 Chromatin conformation capture (3C) fragment interactions.
