# Supplementary figures and images for: Mapping of Human FOXP2 Enhancers Reveals Complex Regulation
Source: Front Mol Neurosci. 2018 Feb 21;11:47. doi: 10.3389/fnmol.2018.00047 (PMC5826363; doi:10.3389/fnmol.2018.00047)

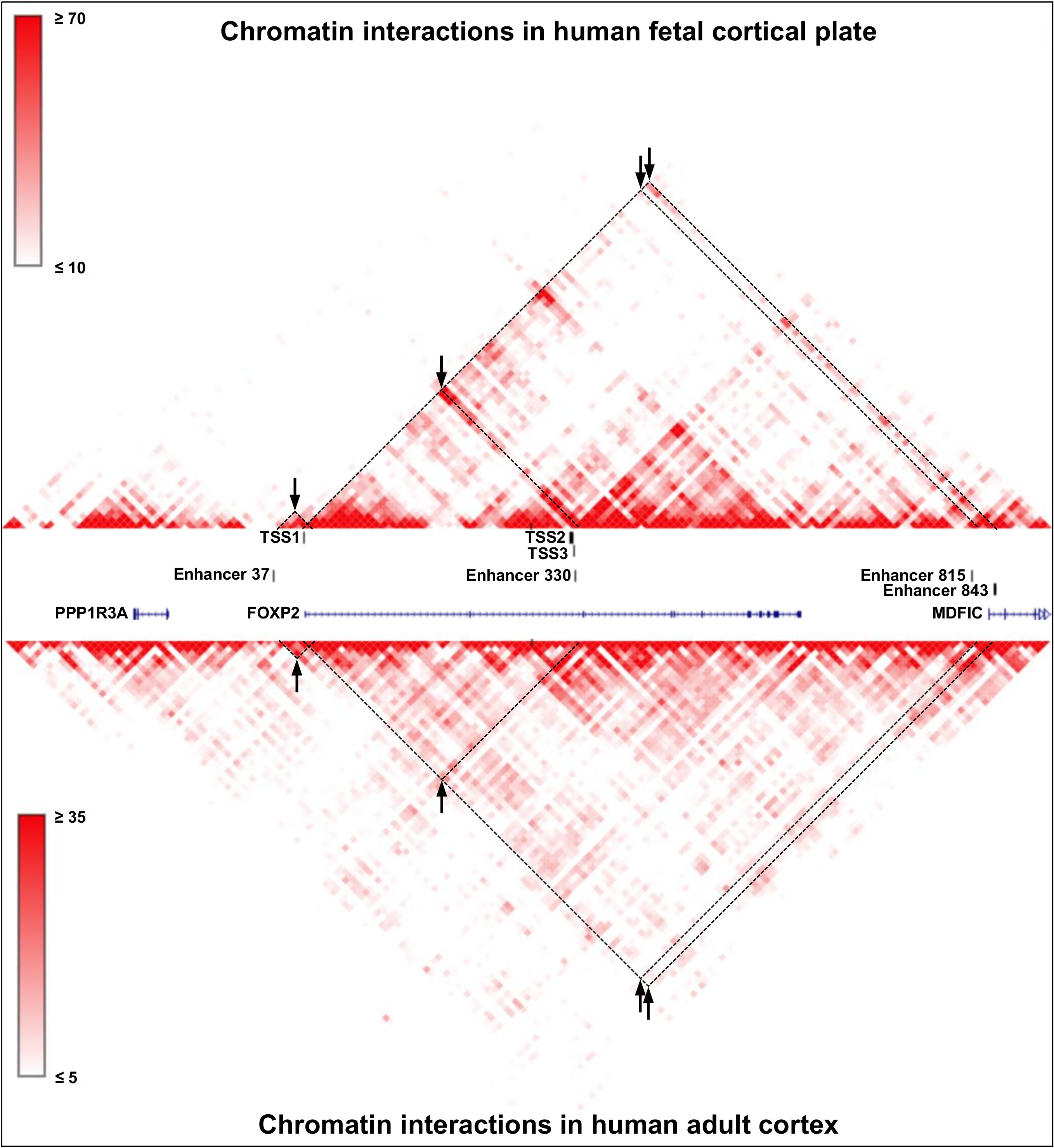

Supplement: FIGURE S1 — Chromatin interactions obtained from publicly available Hi-C data in neuronal tissue samples. Chromatin interactions at chromosome 7 between nucleotide 113350000 and 114640000 (hg19) determined via Hi-C were visualized in the “Compare Hi-C” browser application (http://promoter.bx.psu.edu/hi-c/compare.php; last accessed on 05.01.2018) supplied by the YUE lab (Wang et al., unpublished). The middle panel displays the location of FOXP2 and the neighboring genes PPP1R3A and MDFIC. The position of the FOXP2 promoters at TSS1, TSS2, and TSS3 and the putative enhancers -37, 330, 815, and 843, are annotated. The top panel shows the heat map of chromatin interactions in a sample of human fetal cortical plate tissue (Won et al., 2016) and the bottom panel shows the heat map of chromatin interactions in a sample of human adult cortex tissue (Zhang et al., unpublished). Color intensity indicates the strength of the measured chromatin interaction between two distant regions at a resolution of 10 kb. In the fetal cortical plate, TSS1 of FOXP2 shows stronger interactions to genomic regions at enhancers -37, 330, and 843 than to other regions in this genomic locus (black arrows). In the adult cortex, the chromatin interactions between TSS1 and these regions were generally weaker (black arrows). The dashed lines indicate the interaction between 10 kb blocks containing the elements of interest. [file Image_1.tif]
