# Supplementary material for: Mapping of Human FOXP2 Enhancers Reveals Complex Regulation
Source: Front Mol Neurosci. 2018 Feb 21;11:47. doi: 10.3389/fnmol.2018.00047 (PMC5826363; doi:10.3389/fnmol.2018.00047)
Supplement: TABLE S1 — ChIP-qPCR Primers. [file Table_1.pdf]

Table S1: ChIP-qPCR Primers

ID	Forward primer	Reverse primer
1	GTTCAAGTTGGCCGTTCTTTC	TGAAACTTTCCTCCAAATAGCTG
2	GCGAGCAGTGTGTGTACAGTTC	GTGTGTCTGTGTGTGTGAATGTG
3	TGGCCTGGTTTTACTTTTATTCC	AGGTAACAGCCAAGTGAGAAGC
4	AAGGTGAAATGTGAAATTTGCTG	ACATCTGCCACTACTTTTGTTGC
5	TGGTGCTTTTGTCTCTCTCTCTC	GGTGACAAAGGTTCAAGACTGAG
6	AGCTTGGCTGGGCTATAAATTAC	GTCATCAGGCAATTACTGTAGCC
7	TTGCAAGTCCTGTAATGATGATG	GTGATATTGAAACAGCCCAATTC
8	TGTATTTAGTGTTTGTGTGCATCG	AATGCAAGCTTCCTATCTCTTCC
9	GATTAGCATCTGGATGAAAGCTG	ACACGTTACACCTTCACACTTCC
10	AAGTTAAAGCAAATGCCCACAC	GATTATTCTTGAAAACAATGCCTAAAG
GAPDH promoter	CGGCTACTAGCGGTTTTACG	GCTGCGGGCTCAATTTATAG
Myoglobin Exon2	CAGACGTTCAGCACCAACTG	ACCCAGTGAGCCCATACTTG
