# Supplementary material for: Mapping of Human FOXP2 Enhancers Reveals Complex Regulation
Source: Front Mol Neurosci. 2018 Feb 21;11:47. doi: 10.3389/fnmol.2018.00047 (PMC5826363; doi:10.3389/fnmol.2018.00047)
Supplement: TABLE S2 — Primers used for 3C-qPCR. [file Table_2.pdf]

Table S2: Primers used for 3C-qPCR

Chromosome	Start [hg19]	End [hg19]	3C fragment	Sequence
chr7	113619659	113619682	-107	TTCTTTCAGTACCACTGCTAGGTG
chr7	113652257	113652276	-74	TTCTCTCCCCTCCCCATATC
chr7	113658218	113658234	-68	CCACATGGGAGGAGCAAG
chr7	113667023	113667041	-59	CCCCATTAAATGCTTGCAG
chr7	113686885	113686904	-39	GTTCCTTTTCTGGCAGGATG
chr7	113688356	113688375	-38	CAATGCCTTGGTCATTGTTG
chr7	113689702	113689721	-37	CACACTGATGGATGGCTCAC
chr7	113706877	113706896	-19	GCAAGCCAAAATTCCAAATC
chr7	113708164	113708186	-18	TTGCTCATAGACACAAATCAAGG
chr7	113710846	113710867	-15	AAAACCAGGAAACTGACATTGG
chr7	113715771	113715790	-11	GGGTCTGCTTCCAAGTTCAG
chr7	113721054	113721074	-5	TCTTGCATCAGGAAATTCCAC
chr7	113732140	113732161	Anchor point	TGAAAAGCACATTGCTGTGAAG
chr7	113739754	113739773	13	CTCCCTTGGACACCAGAATC
chr7	113743011	113743032	17	TTGTTCAGTGCTTGACTTTTGG
chr7	113751593	113751615	25	AAGGGAACTTATCCAGCTACAGG
chr7	113755630	113755649	29	GCCAGGAATATTGGCTGTTG
chr7	113764551	113764570	38	TCCAATGGTGCTCACAAAAG
chr7	113772692	113772711	46	TAGATGGAATGGCCAGGAAG
chr7	113779379	113779407	53	GTTGTTTTCGGGGAGGTTG
chr7	113796607	113796626	70	CCACCTTTCAAGACCAGACC
chr7	113803849	113803870	78	CTGGAATGTTACCTAGGGCATC
chr7	113815841	113815863	89	CGATTCTCTTCCCTGTAGAATCC
chr7	113978968	113978990	253	TTCCATGCTCTGGAGATTAAAAC
chr7	113988151	113988176	262	GAGAATTACGTGGTAAATCACAGTTG
chr7	113998725	113998744	272	AGAGACTGGAGGCATTTTGC
chr7	114034128	114034148	308	TCCAAAAGCAATTAGCACACC
chr7	114055582	114055601	329	GGGAGACCAGACACAGGAAC
chr7	114056404	114056423	330	TTCCCAGTGACATGAGCAAG
chr7	114061389	114061413	335	TTTCATTGGCTTTACTTATGTTTCC
chr7	114068822	114068845	342	TGATGCAGTTATTGCCTTTAATTC
chr7	114072519	114072541	346	AACTAAGGTTTTGCAGCTGACTG
chr7	114079808	114079827	353	ATGTGGAGACAGGGTTTTGG
chr7	114329855	114329874	604	GGAAGAGCCAGTGATTGCAG
chr7	114329855	114329874	621	TTGGAAAGGGCTACAGTGACA
chr7	114329855	114329874	633	CACCCATTTCCTCTTTTTCCA
chr7	114432476	114432495	706	GAAGTTGACCCACCACTCCA
chr7	114463228	114463247	737	TGGGCCTCTGTTGTCTCAGT
chr7	114478670	114478689	752	GCAGAACAAGGGTGGACAAA
chr7	114498528	114498547	772	AGCTTTTCTGGGGAGCTGAC
chr7	114535036	114535055	809	TCCTTTAGCGCTGCTCTGAT
chr7	114549286	114549307	823	CAAGCATTTGTCCTTTCTTTGA
chr7	114568978	114568997	843	CAGCTGTGAGAACCCCTGAG
chr7	114590707	114590726	864	CAGGGGAGGTAAAGGCTCAA
chr7	115112801	115112820	1386	CACAAGGGCCCAAGTTACAA
chr7	115117519	115117538	1391	GGAGATCCCGAGAAAAGCTC
