# Supplementary material for: Mapping of Human FOXP2 Enhancers Reveals Complex Regulation
Source: Front Mol Neurosci. 2018 Feb 21;11:47. doi: 10.3389/fnmol.2018.00047 (PMC5826363; doi:10.3389/fnmol.2018.00047)
Supplement: TABLE S3 — Primers used to clone promoter and enhancer regions. [file Table_3.pdf]

Table S3: Primers used to clone promoter and enhancer regions

Reg. Element	Orientation	Start [hg19]	End [hg19]	Sequence
TSS1	Forward	113724817	113724838	ATCAGAGCTCGTTCAAGTTGGCCGTTCTTTCC
	Reverse	113726588	113726609	ATCACTCGAGAAGCCCCCAAACAACACACC
TSS2	Forward	114051220	114051242	ATCAGAGCTCCTCACATCGCCAAAGGTATACC
	Reverse	114055298	114055324	ATCACTCGAGGCACAGAAAACAGTATAAAAAGAAGGG
TSS3	Forward	114055454	114055475	ATCAGAGCTCTAGAGAGGGGTGGGATTTTACG
	Reverse	114056438	114056459	ATCACTCGAGGGTCAGAGGAAAAAGCTGTTGG
Element 37	Forward	113688009	113688029	ATCAGGTACCCACAGGGGAACCTCTGCAAAACTGC
	Reverse	113475999	113476018	ATCACTCGAGCACAATTTGTGGTGGGTGGGTGGG
Element 330	Forward	114056847	114056867	ATCAGGTACCGGTTTTCACGGAAAGCAGAGT
	Reverse	114058626	114058647	ATCAGAGCTCGACGTCTGTTCTGTGAGTTTCC
Element 815	Forward	114541369	114541389	ATCAGGTACCAGTATTGGCACACCCAGCAA
	Reverse	114542182	114542201	ATCAGAGCTCTGTTCCTAGGCGTGTCTGTG
Element 843	Forward	114568454	114568473	ATCAGGTACCAGTGTGATGGCAGAACATGG
	Reverse	114572392	114572411	ATCACTCGAGACAACTCCCCAATTTCTCGC
